# Fundamental differences

**DOI:** 10.7554/eLife.34477

**Published:** 2018-02-01

**Authors:** Peter W Gunning, Edna C Hardeman

**Affiliations:** School of Medical SciencesUNSW SydneySydneyAustralia

**Keywords:** actin, coding sequence, isoforms, Mouse

## Abstract

The differences between β- and γ-actin are deeper than those between the amino acid sequences of these two proteins.

**Related research article** Vedula P, Kurosaka S, Leu NA, Wolf YI, Shabalina SA, Wang J, Sterling S, Dong D, Kashina A. 2017. Diverse functions of homologous actin isoforms are defined by their nucleotide, rather than their amino acid sequence. *eLife* **6**:e31661. doi: 10.7554/eLife.31661

Protein isoforms – proteins that are similar to each other and perform similar roles within cells – have played an important role in the generation of biological diversity throughout evolution. In some cases a single gene can encode two or more isoforms by exploiting a process called alternative splicing. In other cases two or more closely related genes are responsible for the isoforms. At its simplest, isoform generation provides a mechanism to specialize the properties of a gene or protein at one of three levels – DNA, messenger RNA (mRNA) or the sequence of amino acids in the protein itself (Figure 1). Depending on biological context, any one of these levels may be more or less important than the others, and there is no a priori reason to believe that only one of them will be important (Gunning, 2003).

**Figure 1.**
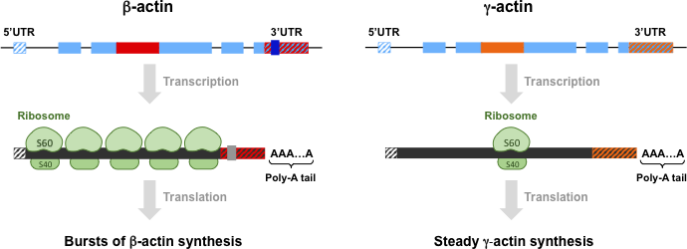
Differences between β-actin and γ-actin at different levels. The genes for β-actin (top left) and γ-actin protein (top right) are similar in many ways, as are the mRNAs and proteins they code for, but the small differences between them are important. At the level of genes, both intron 3 (shown in red for β-actin and orange for γ-actin) and the 3´ untranslated region (3´ UTR) are different: moreover, these two elements are conserved features that are found in both birds and mammals. β-actin also has four codons (not shown) that code for four amino acids that are not found in the γ-actin gene or protein. (Exons, the regions of DNA that are transcribed to produce mRNA, are shown in pale blue). At the level of mRNA, β-actin has a high density of ribosomes (shown in green), whereas γ-actin has a low density; β-actin also has a sequence (grey) that directs the mRNA molecules to the periphery of the cell. At the level of proteins, it has been predicted that β-actin is produced in rapid bursts, whereas γ-actin is produced steadily at a low rate. The essential nature of the differences between β-actin and γ-actin at these three levels is the subject of intensive ongoing research. We thank Yao Wang for preparing this figure.

Actins are proteins that are essential for a number of fundamental cellular processes such as cell division and muscle contraction. In mammals, there are six actin isoforms, and two of these – β-actin and γ-actin – have been the focus of much research over the past 40 years. It is not in dispute that β-actin and γ-actin perform different functions in mammalian cells, and engage in distinct biological processes (Dugina et al., 2009; Patrinostro et al., 2017; Tondeleir et al., 2013; Müller et al., 2013). Moreover, β-actin is essential for survival, but γ-actin is not (Perrin and Ervasti, 2010). However, despite all these differences, the two isoforms differ by only four amino acids (Vandekerckhove and Weber, 1978).

The fact that β-actin and γ-actin are both conserved across birds and mammals is clear evidence for selective pressure to maintain certain features of these two proteins. However, it is not clear why birds and mammals have evolved to use two near-identical proteins to build cell architecture (Ampe and Van Troys, 2017). The nucleotide sequence of the β-actin gene, independent of the encoded protein, has also been conserved in evolution (Ng et al., 1985). For example, the 3´ untranslated region (3´UTR) of this gene is highly conserved among birds and mammals and is unique to β-actin mRNA. Intron 3 is similarly conserved among birds and mammals. Could it be that the nucleotide sequence of the β-actin gene – in particular, regions of the gene that do not code for protein products – contributes to its essential role?

Now, in eLife, Anna Kashina and co-workers at the University of Pennsylvania and the National Institutes of Health – including Pavan Vedula and Satoshi Kurosaka as joint first authors – report that the differences between β-actin and γ-actin at the level of nucleotides may be as important as the differences at the level of amino acids (Vedula et al., 2017). In a elegant series of experiments on mice Vedula et al. used genetic tools to edit the four codons that are found in the β-actin gene but not in the γ-actin gene. This meant that the mutant mice produced γ-actin rather than β-actin. Since β-actin is essential for survival, whereas γ-actin is not, one might have expected the mutant mice to die, but they did not. Indeed, the mutant mice were indistinguishable from wild type mice in terms of embryology, survival, tissue histology and various actin-dependent processes (such as the properties of embryonic fibroblasts). The inescapable conclusion is that there are one or more regions of the nucleotide sequence of the β-actin gene – besides the regions that code for the four extra amino acids – that have roles that are essential for survival (Figure 1).

Based on current knowledge, one might expect one of these roles to be localizing molecules of β-actin mRNA (but not γ-actin mRNA) to the periphery of cells. However, Vedula et al. draw attention to another feature unique to the β-actin mRNAs: they have a much higher density of ribosomes than γ-actin mRNAs. This does not relate to the relative amounts of the two proteins in cells, but to a large difference in the capacity of cells to make these proteins at any point in time. The fact that a cell can produce a large amount of β-actin protein in a short burst, whereas it would take a longer time to produce the same amount of γ-actin, could be significant. They also report differences in ribosome density between other actin isoforms and isoforms of histones and tubulins.

In the mid-1970s the Nobel Prize-winning biologist Francois Jacob eloquently argued that much of evolution is 'tinkering' (Jacob, 1977). But would Jacob have predicted that tinkering at the level of the nucleotides in the gene sequence could, as the results of Vedula et al suggest, be as important as tinkering at the level of the amino acids in proteins?
